# Identification and Verification of Necroptosis-Related Gene Signature and Associated Regulatory Axis in Breast Cancer

**DOI:** 10.3389/fgene.2022.842218

**Published:** 2022-02-16

**Authors:** Ting Hu, Xiangwang Zhao, Yanxia Zhao, Jing Cheng, Jie Xiong, Chong Lu

**Affiliations:** ^1^ Cancer Center, Union Hospital, Tongji Medical College, Huazhong University of Science and Technology, Wuhan, China; ^2^ Department of Breast and Thyroid Surgery, Union Hospital, Tongji Medical College, Huazhong University of Science and Technology, Wuhan, China

**Keywords:** breast cancer, necroptosis, bioinformatics analysis, prognostic biomarker, BCL2

## Abstract

**Background:** Breast invasive carcinoma (BRCA) is the second leading cause of malignancy death among women. Necroptosis is a newly discovered mechanism of cell death involved in the progression and prognosis of cancer. The role of necroptosis-related genes (NRGs) in BRCA is still a mystery.

**Methods:** LASSO Cox regression analysis was performed to construct a prognostic necroptosis-related signature. A ceRNA was constructed to explore the potential lncRNA-miRNA-mRNA regulatory axis in BRCA.

**Results:** A total of 63 necroptosis-related genes were differentially expressed in BRCA. We also summarized the genetic mutation landscape of NRGs in BRCA. BRCA patients with low expression of BCL2 and LEF1, as well as high expression of PLK1 and BNIP3, had a poor OS, DSS, and DFS. A necroptosis-related prognostic signature with four genes (BCL2, LEF1, PLK1, and BNIP3) was constructed, and it could serve as a prognosis biomarker in BRCA, predicting the OS rate with medium to high accuracy. Moreover, the risk score was correlated with immune infiltration in BRCA. Further comprehensive analysis revealed that the expression of BCL2, LEF1, PLK1, and BNIP3 was correlated with tumor mutation burden, microsatellite instability, drug sensitivity, and pathology stage. Previous studies have been extensively studied. The roles of LEF1, PLK1, and BNIP3 in BRCA and BCL2 were selected for further analysis. We then constructed a ceRNA network, which identified an lncRNA LINC00665/miR-181c-5p/BCL2 regulatory axis for BRCA.

**Conclusion:** The bioinformatics method was performed to develop a prognostic necroptosis-related prognostic signature containing four genes (BCL2, LEF1, PLK1, and BNIP3) in BRCA. We also constructed a ceRNA network and identified an lncRNA LINC00665/miR-181c-5p/BCL2 regulatory axis for BRCA. Further *in vivo* and *in vitro* studies should be conducted to verify these results.

## Introduction

Breast cancer is the most common malignancy and the second leading cause of cancer death among women (DeSantis et al., 2017). Furthermore, the incident rates of breast cancer have increased over the past 15 years (DeSantis et al., 2016). Despite a significant decrease in breast cancer-associated deaths due to the progress in early detection and therapy, the overall prognosis of breast cancer patients remains poor, especially of triple-negative breast cancer patients (Foulkes et al., 2010; Hart et al., 2016). Thus, the identification of novel biomarkers for prognosis and drug screening in invasive breast cancer, providing additional strategies for predicting survival and optimizing treatments, is of great significance.

Necroptosis is a newly discovered mechanism of cell death mediated by RIP1, RIP3, and MLKL (Zhe-Wei et al., 2018; Gong et al., 2019). Increasing studies suggest that necroptosis is implicated in the pathogenesis of various diseases, including Parkinson’s, Alzheimer’s, vascular atherosclerosis, and infectious diseases (Zhe-Wei et al., 2018; Yuan et al., 2019; Wu et al., 2020). Recent evidence has also indicated that necroptosis could accelerate cancer metastasis and T-cell death (Najafov et al., 2017). With the characteristics of both necrosis and apoptosis, necroptosis may trigger and amplify antitumor immunity in the immunotherapy of malignancy (Gong et al., 2019). Moreover, some necroptosis regulators may serve as prognosis markers for cancer and other diseases (Zhang et al., 2018; Park et al., 2020). Thus, necroptosis-related genes may also be of vital significance in the prognosis of BRCA.

With the development of the Cancer Genome Atlas (TCGA), big data mining has been suggested as one of the promising ways to study the tumorigenesis mechanism and associated prognosis marker and therapy target of cancer. Herein, we mined a database to investigate the expression profiles and prognosis significance of necroptosis-related genes and potential regulatory axis in BRCA.

## Materials and Methods

### Dataset and Preprocessing

Based on previous reports about necroptosis, a total of 67 necroptosis-related genes were obtained (Supplementary Table S1). The gene expression profile of necroptosis-related genes in breast invasive carcinoma (BRCA) was isolated from the Cancer Genome Atlas (TCGA) database (https://cancergenome.nih.gov/). A total of 1209 BRCA samples, including 113 normal samples and 1096 breast cancer samples, and the corresponding clinical characteristics of BRCA patients were downloaded for further analysis. The mRNA level of necroptosis-related genes was analyzed and visualized by R (version 4.0.3) with ggplot2 packages with the Student t-test.

### Genetic Mutation, GO, and KEGG Pathway

We obtained genetic mutation data from the TCGA BRCA dataset. We used the “maftools” package in R software to show the genetic mutation of differentially expressed necroptosis-related genes (DENRGs). Functional enrichment analysis, including gene ontology (GO) analysis and Kyoto Encyclopedia of Genes and Genomes (KEGG) pathways analysis, was performed using the cluster profiler package in R.

### Development of Necroptosis-Related Prognostic Gene Signature

The Kaplan–Meier methods were applied to identify the prognostic necroptosis-related gene with the calculation of the *p*-values, hazard ratio (HR), and 95% confidence interval (CI) using the log-rank test. We then performed LASSO cox regression analysis to construct a necroptosis-related prognostic gene signature using prognostic necroptosis-related genes. A computational equation (sum of coefficients x necroptosis-related gene expression) was used to calculate the risk score of each BRCA patient. All BRCA samples were separated into two subgroups with the median value of the risk score as the cutoff value. The Kaplan–Meier analysis was applied to draw the OS curve, and a Time ROC analysis was conducted to evaluate the predictive performance of this prognostic signature. Moreover, the Pearson correlation analysis was performed to evaluate the correlation between the risk score and immune infiltration.

### Construction of a Potential Regulatory Axis

After the hub gene was obtained, we used miRDB (http://mirdb.org/), StarBase (http://starbase.sysu.edu.cn/), and miRWalk (http://mirwalk.umm.uni-heidelberg.de/) to identify the miRNA target of the hub gene. This was followed by using StarBase (http://starbase.sysu.edu.cn/) and the LncBase module of the DIANA tool (http://carolina.imis.athena-innovation.gr/) to predict lncRNA targets interacting with miRNA. Moreover, the expression of miRNA and lncRNA was detected with a Student t-test using the TCGA BRCA dataset. A *p*-value of < 0.05 was considered a statistically significant difference.

### Human Tissues and Quantitative Real Time-Polymerase Chain Reaction

We obtained 52 breast cancer tissues and normal breast tissues from patients who underwent a breast cancer removal in Union Hospital of Tongji Medical College. The studies involving human participants were reviewed and approved by the Ethics Committee of Union Hospital of Tongji Medical College. The patients/participants provided their written informed consent to participate in this study. Histological diagnosis and tumor grade were assessed by three experienced pathologists in accordance with the 2010 American Joint Committee on Cancer staging system.

The total RNA of breast cancer tissues and normal tissues was extracted with the TRIzol reagent (Vazyme, Nanjing, China). The synthesis of cDNAs corresponding to the mRNAs of interest depended on PrimeScript RT-polymerase (Vazyme) and SYBR-Green Premix (Vazyme) with specific PCR primers (Sangon Biotech Co., Ltd, Shanghai, China). The specific primers used were as follows: GAPDH-F: GGG​AAG​GTG​AAG​GTC​GGA​GT; GAPDH-R: GGG​GTC​ATT​GAT​GGC​AAC​A; BCL2-F: ACT​GGC​TCT​GTC​TGA​GTA​AG; BCL2-R: CCT​GAT​GCT​CTG​GGT​AAC. The fold-changes were calculated with the 2^−ΔΔCt^ method. The difference in BCL2 expression and the prognosis of BCL2 in breast cancer were evaluated with Student’s t-test and the Kaplan–Meier analysis in GraphPad Prism 7 software (GraphPad, Inc., La Jolla, CA, United States).

## Results

### The Expression of Necroptosis-Related Genes in BRCA

**FIGURE 1:**
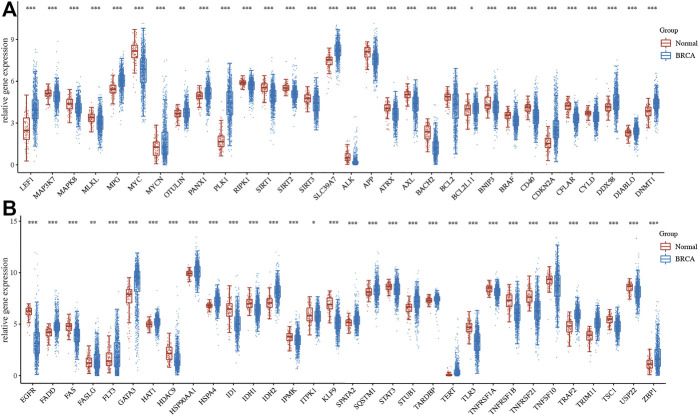
Gene expression of necroptosis-related gene in BRCA. **(A–B)** The mRNA level of necroptosis-related genes in BRCA *versus* breast tissues. *p < 0.05; **p < 0.01; ***p < 0.001.

Among 67 necroptosis-related genes, a total of 63 necroptosis-related genes were differentially expressed in breast cancer with a fold change of 2 or <0.5 and a *p*-value of 0.05, which was shown in Figures 1A,B. To be more specific, 35 necroptosis-related genes were downregulated, while 28 necroptosis-related genes were upregulated in BRCA.

### Genetic Mutation and Functional Enrichment Analysis of DENRGs

**FIGURE 2:**
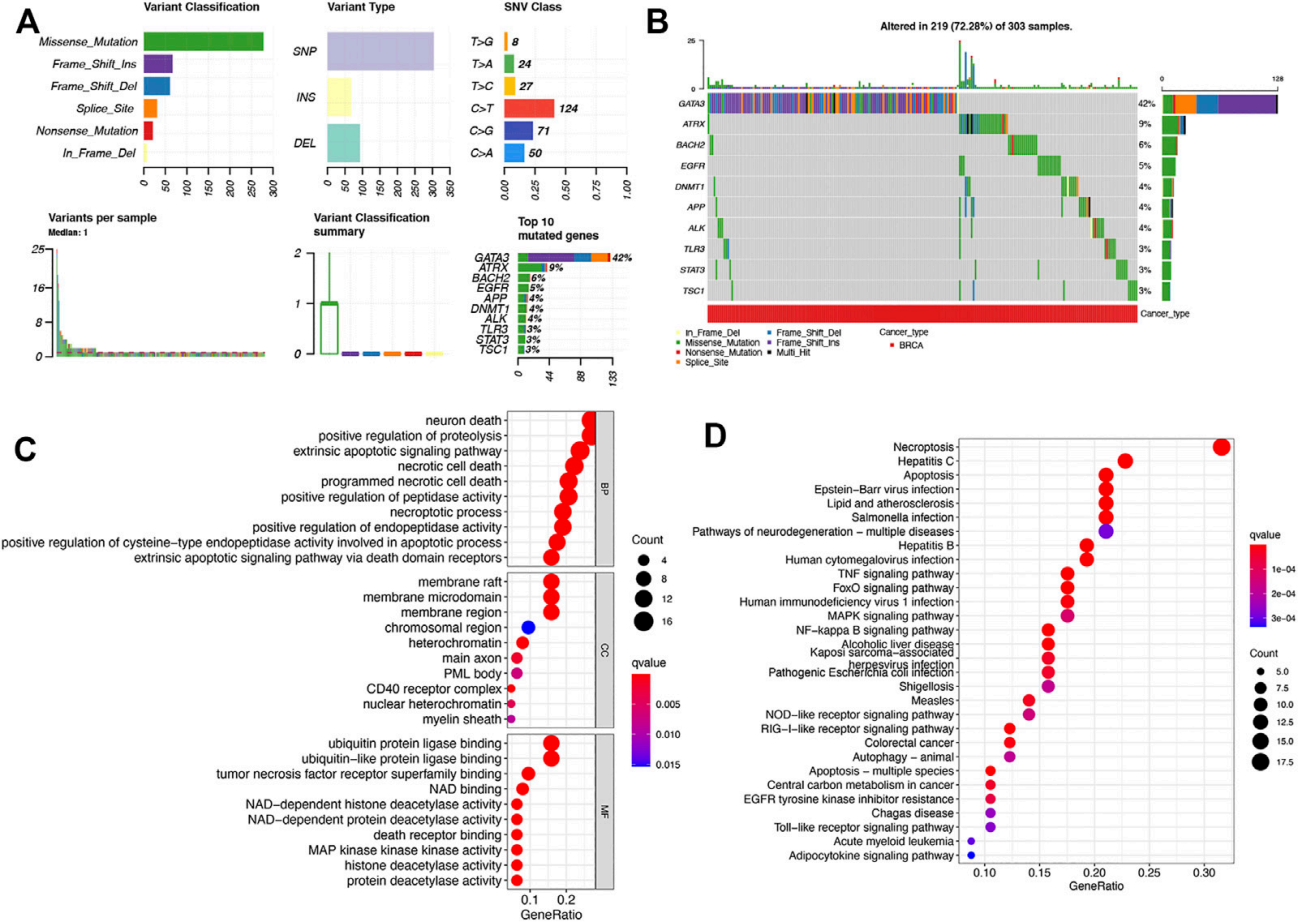
Genetic mutation and functional enrichment analysis of necroptosis-related gene in BRCA. **(A–B)** The genetic mutation of necroptosis-related genes in BRCA. **(C–D)** The enriched items in gene ontology and Kyoto Encyclopedia of Genes and Genomes analysis. BP, biological process; CC, cellular component; MF, molecular function.

Figures 2A,B revealed simple nucleotide variation of DENRGs in BRCA cases, suggesting that 219 of 303 (72.28%) BRCA samples presented with simple nucleotide variation and GATA3 was the gene with the highest frequency of mutation, followed by ATRX and BACH2. We found that missense mutation ranked the top variant classification and C > T was the most common SNV class (Figure 2A). The result of GO analysis revealed that the DENRGs were mainly associated with positive regulation of proteolysis, necrotic cell death and process, ubiquitin protein ligase binding, and death receptor binding (Figure 2C). Moreover, the result of KEGG pathway analysis revealed that these DENRGs are mainly associated with necroptosis, apoptosis, TNF signaling pathway, MAPK signaling pathway, NF-kappa B signaling pathway, NOD-like receptor, and Toll-like receptor signaling pathway (Figure 2D).

### The Prognostic Value of Necroptosis-Related Genes in Breast Cancer

**FIGURE 3:**
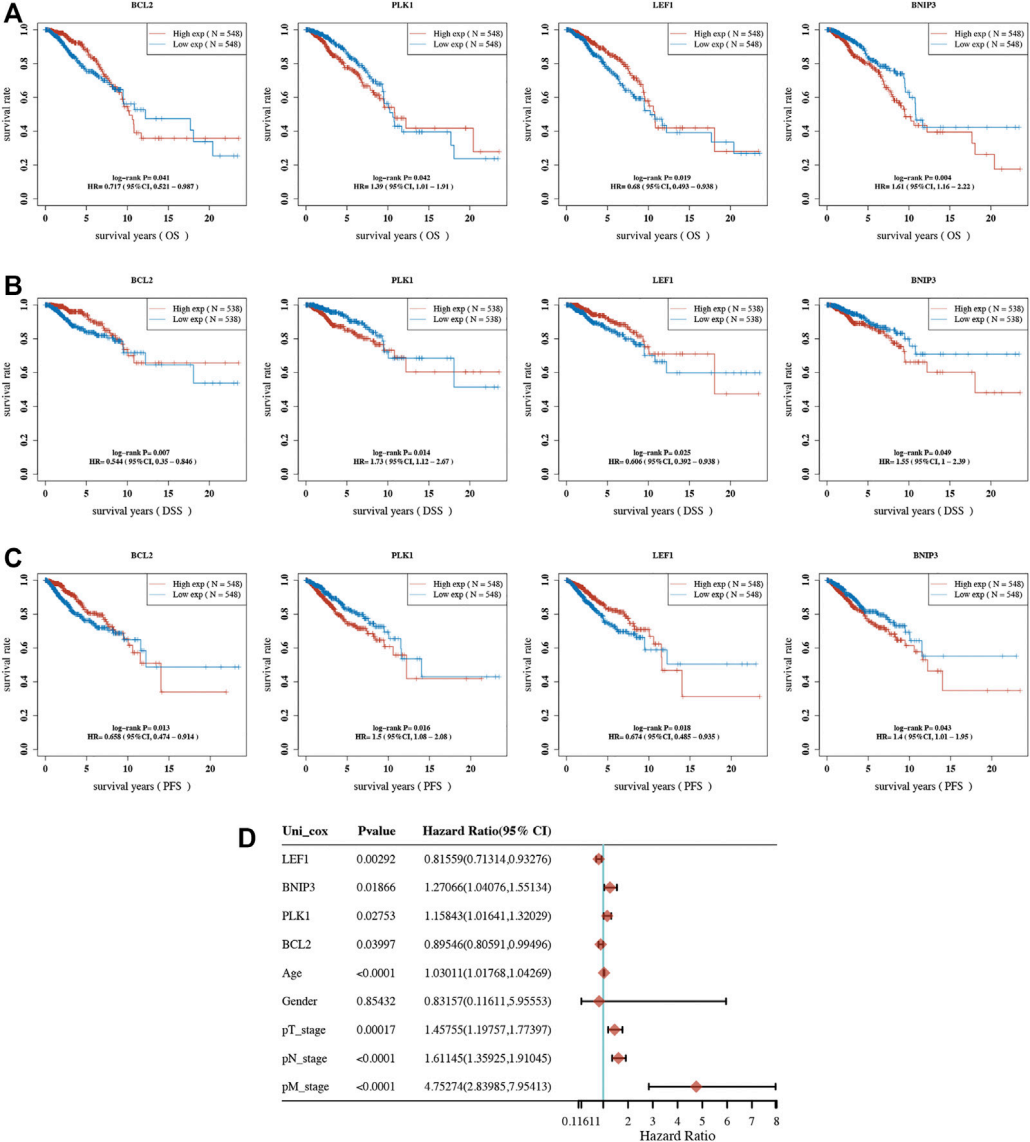
Prognostic analysis of necroptosis-related gene in BRCA. OS **(A)**, DSS **(B),** and PFS **(C)** curve in BRCA patients with high/low expression of BCL2, PLK1, LEF1, and BNIP3. **(D)** Univariate survival analysis considering the expression of BCL2, PLK1, LEF1, and BNIP3 as well as clinical characters in BRCA. OS, overall survival; DSS, disease-specific survival; PFS, progression-free survival.

Overall survival (OS) analysis, disease-specific survival (DSS) analysis, and progression-free survival (PFS) analysis were performed to analyze the prognosis value of necroptosis-related genes in BRCA. As a result, a total of 7 necroptosis genes (BCL2, BNIP3, FASLG, HSP90AA1, LEF1, PANX1, and PLK1) with prognostic significance in OS analysis (Table 1), 9 necroptosis genes (BCL2, BNIP3, FASLG, IDH2, LEF1, MYCN, PLK1, SLC39A7, and TLR3) with prognostic significance in DSS analysis (Table 2), and 9 necroptosis genes (BCL2, FASLG, FLT3, BNIP3, LEF1, PLK1, SLC39A7, TNFRSF1B, and TNFRSF21) with prognostic significance in PFS analysis were obtained (Table 3). Interestingly, BCL2, LEF1, PLK1, and BNIP3 had prognostic values in all OS, PFS, and DFS analyses. To be more specific, BRCA patients with low expression of BCL2 and LEF1, as well as high expression of PLK1 and BNIP3, had a poor OS (Figure 3A), DSS (Figure 3B), and DFS (Figure 3C). Moreover, univariate survival analysis revealed that BCL2, PLK1, PEF1, BNIP3, age, pT stage, pN stage, and pM stage were independent factors affecting the prognosis of BRCA patients (Figure 3D). Thus, we selected BCL2, LEF1, PLK1, and BNIP3 for further study.

**TABLE 1:** Necroptosis genes with prognostic significance in overall survival analysis.

Genes	p value	HR	Low 95% CI	High 95% CI
BCL2	0.041440897	0.717488382	0.52146467	0.98719934
BNIP3	0.004046459	1.608510841	1.163256858	2.224192453
FASLG	0.012675752	0.660756765	0.477030986	0.915243485
HSP90AA1	0.014627108	1.486360425	1.081301896	2.043154943
LEF1	0.018745669	0.680143707	0.493194005	0.937958405
PANX1	0.042556987	1.398883286	1.011330128	1.934951203
PLK1	0.042378952	1.39031232	1.011398819	1.911183118

**TABLE 2:** Necroptosis genes with prognostic significance in disease-specific survival analysis.

Genes	p value	HR	Low 95% CI	High 95% CI
BCL2	0.00690657	0.54379301	0.34952248	0.84604241
BNIP3	0.04931194	1.54570952	1.0013124	2.38608643
FASLG	0.02829859	0.61098492	0.39337684	0.94896938
IDH2	0.04160063	1.58087688	1.01757946	2.45599659
LEF1	0.02460085	0.60602134	0.39157325	0.93791357
MYCN	0.01084538	1.7931582	1.14420414	2.81017715
PLK1	0.01419105	1.72641859	1.11588039	2.67100416
SLC39A7	0.024655	1.63657472	1.0649007	2.51514233
TLR3	0.03486718	0.62873254	0.4085576	0.9675615

**TABLE 3:** Necroptosis genes with prognostic significance in progression-free survival analysis.

Genes	p value	HR	Low 95% CI	High 95% CI
BCL2	0.01259054	0.658426309	0.474179309	0.914264279
FASLG	0.034616286	0.700502977	0.503505242	0.97457659
FLT3	0.014722362	0.66642712	0.480982722	0.923370187
BNIP3	0.042662824	1.40475503	1.011302535	1.951282259
LEF1	0.018237128	0.673746935	0.485412444	0.935153059
PLK1	0.015776569	1.49606572	1.078718396	2.074881311
SLC39A7	0.049666391	1.385389836	1.000475437	1.918392923
TNFRSF1B	0.022579132	0.679879393	0.487994768	0.947215052
TNFRSF21	0.032152263	1.433905627	1.031183664	1.993907991

### Construction of a Necroptosis-Related Prognostic Signature

**FIGURE 4:**
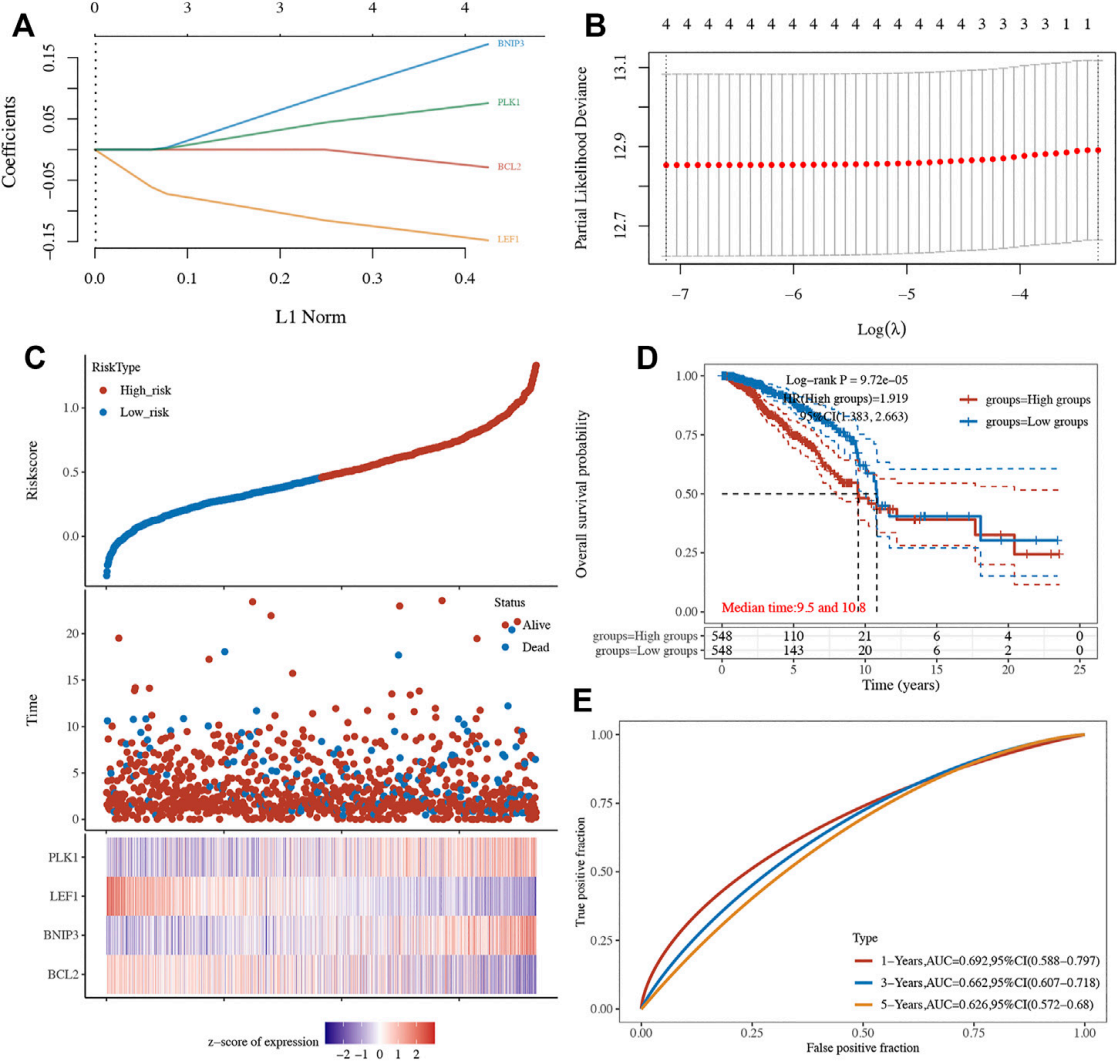
Construction of a necroptosis-related prognostic signature. **(A–B)** The coefficient and partial likelihood deviance of prognostic signature. **(C)** The riskScore distribution, survival status of BRCA cases, and gene expression profile of this prognostic signature. **(D–E)** Overall survival curve in high/low-risk group and the ROC curve evaluating prognosis predicting performance of BRCA patients.

**FIGURE 5:**
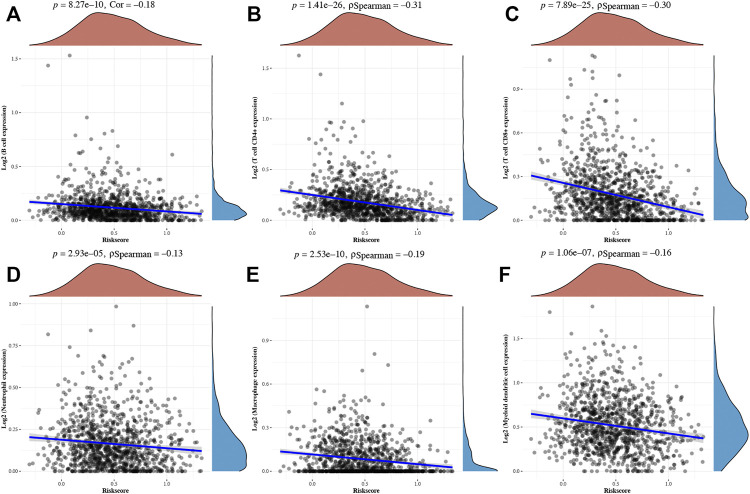
Correlation between riskscore and immune infiltration in BRCA. The correlation between riskscore and the expression of B cells **(A)**, CD4^+^ T cells **(B)**, CD8^+^ T cells **(C)**, neutrophils **(D)**, macrophage **(E)**, and dendritic cells **(F)** in BRCA.

A LASSO cox regression analysis was conducted to develop a necroptosis-related prognostic signature using BCL2, LEF1, PLK1, and BNIP3. The coefficient and partial likelihood deviance of the prognostic signature are shown in Figures 4A,B. Figure 4C shows the riskScore distribution, the survival status of BRCA cases, and the gene expression profile of this prognostic signature. With a formula (Riskscore = (−0.0289) * BCL2 + (0.172) * BNIP3 + (−0.1479) * LEF1 + (0.0757) * PLK1), the risk score of BRCA patients was calculated, and all BRCA cases were divided into high- and low-risk groups. As shown in Figure 4D, BRCA patients with a high risk score had a poor prognosis *versus* low risk score patients, with a median time of 9.5 vs 10.8 years (*p* = 9.75e^−5^). Moreover, the AUC was 0.692, 0.662, and 0.626 in a 1-year, 3-year, and 5-year ROC curve, respectively, which demonstrated that this prognostic signature had good performance in predicting the prognosis of BRCA patients (Figure 4E). Moreover, further analysis revealed that the immune infiltration level of B cells (*p* = 8,27e^−10^, Figure 5A), CD4^+^ T cells (*p* = 1.41e^−26^, Figure 5B), CD8^+^ T cells (*p* = 7.89e^−25^, Figure 5C), neutrophils (*p* = 2.93e^−5^ Figure 5D), macrophage (*p* = 2.53e^−10^, Figure 5E), and dendritic cells (*p* = 1.06e^−7^,Figure 5F) was negatively correlated with the risk score of BRCA patients.

### Comprehensive Analysis of BCL2, LEF1, PLK1, and BNIP3 in BRCA

**FIGURE 6:**
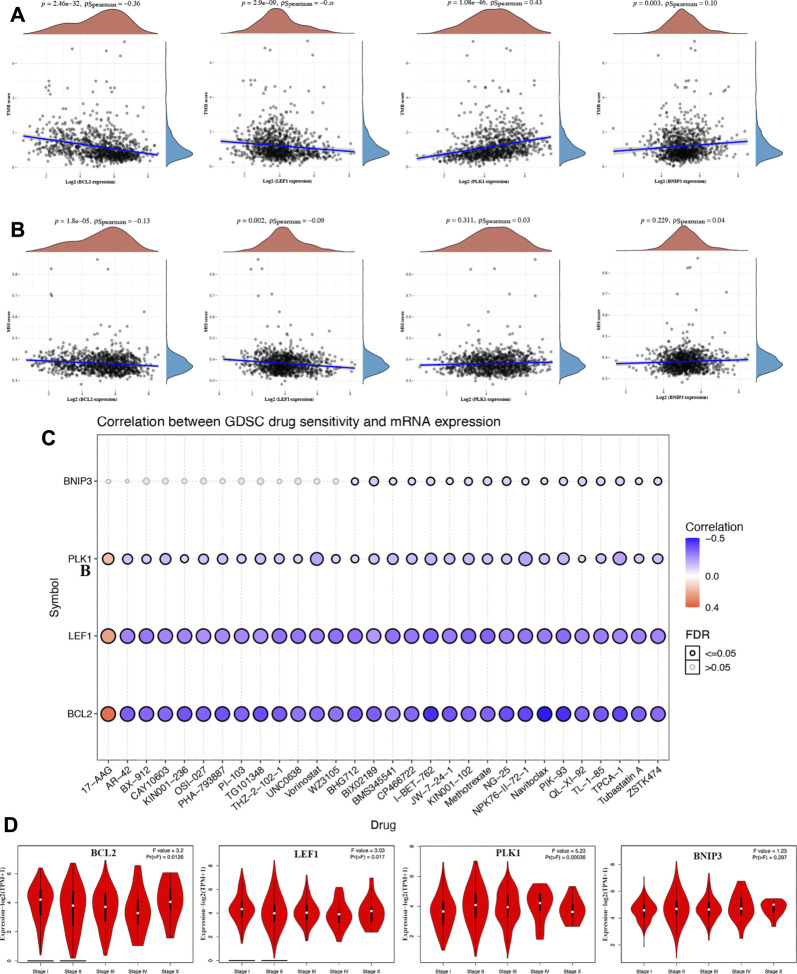
Comprehensive analysis of BCL2, LEF1, PLK1, and BNIP3 in BRCA. The correlation between necroptosis-related genes and TMB **(A)** and MSI. **(B, C)** The correlation between necroptosis-related genes and drug IC50 of GDSC. **(D)** The correlation between necroptosis-related genes and pathology stage in BRCA. TMB, tumor mutation burden; MSI, microsatellite instability; GDSC, genomics of drug sensitivity in cancer.

Increasing evidence suggests TMB as a predictive marker for immunotherapy efficacy in cancer (Liu et al., 2019; Samstein et al., 2019). MSI was also referred as a predictive marker for cancer immunotherapy (Chang et al., 2018). To clarify the important role of BCL2, LEF1, PLK1, and BNIP3 in BRCA, we then explored their correlation with TMB and MSI in BRCA. We found that the TMB score decreased as the expression of BCL2 (*p* = 2.46e^−32^) and LEF1 (*p* = 2.9e^−9^) increased in BRCA (Figure 6A). Moreover, the TMB score was positively correlated with the expression of PLK1 (*p* = 1.08e^−46^) and BNIP3 (*p* = 0.003) (Figure 6A). Similarly, the MSI score decreased as the expression of BCL2 (*p* = 1.8e^−5^) and LEF1 (*p* = 0.002) increased in BRCA (Figure 6B). One of the vital ways to develop a therapy target is to analyze its correlation with exiting drugs. Interestingly, drug sensitivity analysis indicated low expression of BCL2, LEF1, and PLK1 was correlated with drug resistance of GDSC (Figure 6C), suggesting that BCL2, LEF1, and PLK1 may serve as the potential biomarkers for drug scanning. We also explored the expression of PRGs in different pathological stages of BRCA patients. As expected, a significant correlation was obtained between the expression of BCL2, LEF1, and PLK1 and the pathological stage (Figure 6D).

### Construction of an lncRNA-miRNA-mRNA Regulatory Axis

**FIGURE 7:**
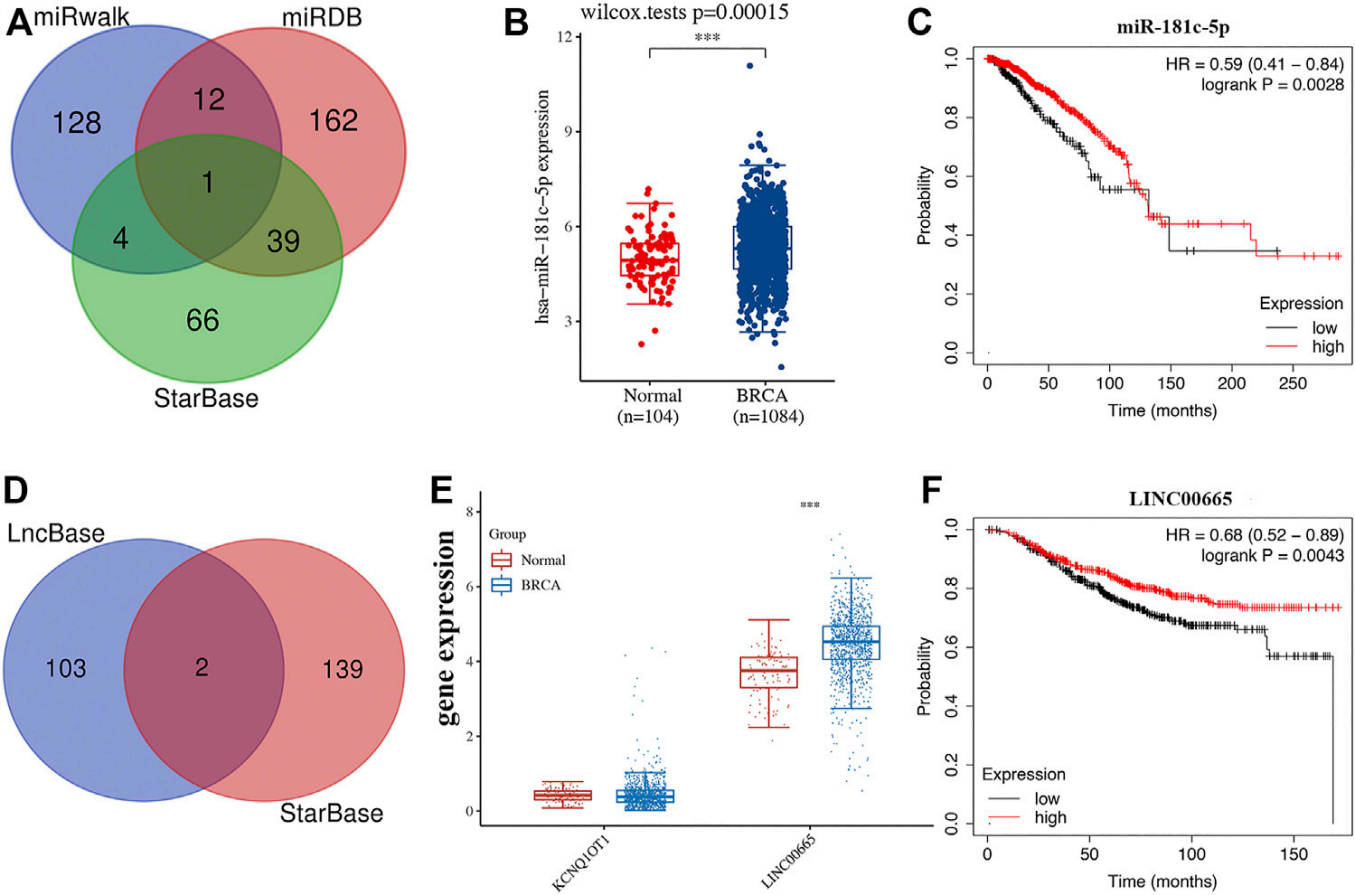
Construction of lncRNA-miRNA-mRNA regulatory axis. **(A)** The miRNA targets predicted by miRDB, miRWalk, and StarBase. **(B–C)** The expression and prognosis value of miR-181c-5p in BRCA. **(D)** The lncRNA targets predicted by lncbase and StarBase. **(E–F)** The expression and prognosis value of lncRNA LINC00665 in BRCA.

Previous studies have been extensively studied. The role of LEF1, PLK1, and BNIP3 in BRCA and BCL2 was selected for further analysis. MiRDB, miRWalk, and StarBase were used to predict the miRNA target of BCL2. As a result, miR-181c-5p was suggested as the miRNA target for BCL2 (Figure 7A). Further studies revealed that miR-181c-5p was upregulated in BRCA (Figure 7B, p = 3e^−5^) and low miR-181c-5p expression was associated with poor survival in BRCA (Figure 7C, *p* = 0.0028). We then detected the lncRNA target of miR-181c-5p, which revealed lncRNA KCNQ1OT1 and LINC00665 as lncRNA targets interacting with miR-181c-5p (Figure 7D). Moreover, we found that LINC00665 was upregulated in BRCA (*p* < 0.001, Figure 7E) and low LINC00665 expression was associated with poor survival in BRCA (Figure 7F, *p* = 0.0043). Thus, the lncRNA LINC00665/miR-181c-5p/BCL2 regulatory axis may play a vital role in the progression of BRCA. Further *in vivo* and *in vitro* studies should be conducted to verify this hypothesis.

### Validation of the Expression and Prognostic Value of BCL2 in BRCA

**FIGURE 8:**
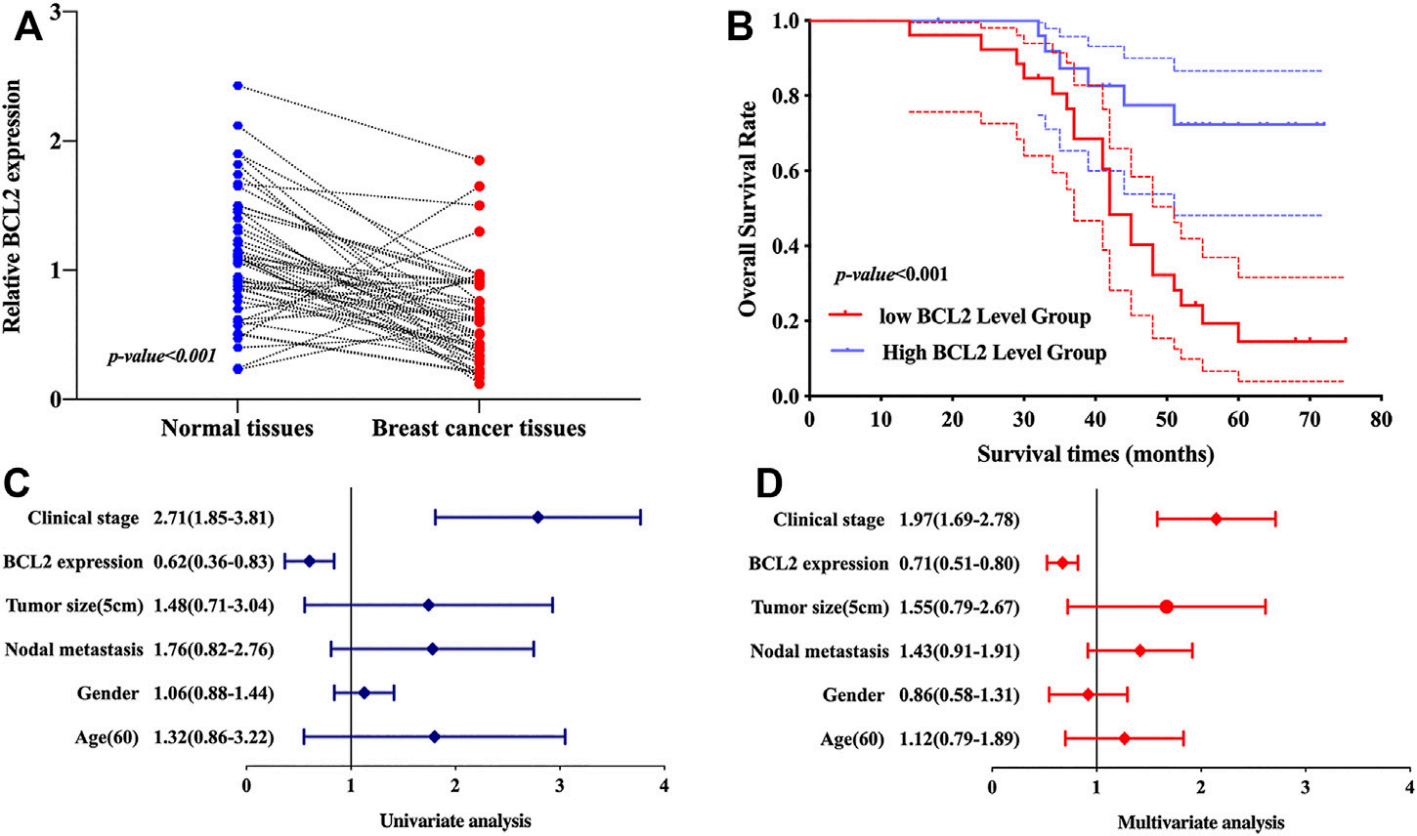
Validation of the expression and overall survival of BCL1 in BRCA. **(A)** The mRNA level of BCL2 was downregulated in BRCA *versus* breast tissues. **(B)** BRCA patients with low BCL2 expression had a poor overall survival. **(E–F)** Univariate and multivariate analyses demonstrated BCL2 and clinical stage as prognosis factors affecting the OS of BRCA patients.

We then verified the expression and prognostic value of BCL2 in BRCA. As expected, the results revealed that BCL2 expression was lower in breast cancer tissues than in normal tissues (Figure 8A, *p* < 0.001). Moreover, prognosis analysis revealed that breast cancer patients with high BCL2 levels had a better overall survival (Figure 8B, *p* < 0.001). Univariate and multivariate analyses revealed that clinical stage and BCL2 expression were factors affecting the prognosis of breast cancer patients (Figures 8C, D). These results were consistent with above data.

## Discussion

Breast cancer still poses a major threat to the health and well-being of American women, being responsible for 30% of all newly diagnosed cancer cases and causing nearly 41,000 deaths each year (Emens, 2018). The overall prognosis of breast cancer patients is poor, especially for triple negative breast cancer patients (DeSantis et al., 2016). Moreover, the mechanisms of tumorigenesis and progression of breast cancer were still unclear. The identification of a novel prognosis signature and clarification of potential mechanisms were critically needed in breast cancer. A previous study revealed that necroptosis-related signature could serve as a prognosis biomarker and predict immune microenvironment infiltration in stomach adenocarcinoma (Wang and Liu, 2021). However, the role of necroptosis in BRCA has not been fully established. In our study, we explored the use of necroptosis-related signatures as a novel biomarker for the prognosis of BRCA.

Expression analysis revealed upregulation of 63 necroptosis-related genes that were differently expressed in BRCA. The results of GO and KEGG revealed that these different expressed necroptosis-related genes were associated with necroptosis, apoptosis, TNF signaling pathway, MAPK signaling pathway, NF-kappa B signaling pathway, NOD-like receptor, and Toll-like receptor signaling pathway. Interestingly, these pathways were involved in the progression of breast cancer. Daintain/AIF-1 promotes tumor cell migration by upregulating TNF-α *via* activating the p38 MAPK signaling pathway in BRCA (Li et al., 2012). Another study revealed that atractylenolide-I could inhibit breast cancer genesis *via* inhibiting the TLR4-mediated NF-κB signaling pathway (Long et al., 2020).

Prognosis analysis revealed that BRCA patients with low expression of BCL2 and LEF1, as well as high expression of PLK1 and BNIP3, have a poor OS, DSS, and DFS. Actually, these genes were suggested as prognosis biomarkers for other types of cancer. PLK1 was suggested as a promising predictive biomarker for diffuse large B-cell lymphoma (Feng et al., 2019). Another study revealed that LEF1 was associated with positive clinical progression, suggesting it as an independent biomarker to predict poor prognosis for nasopharyngeal carcinoma (Zhan et al., 2019).

LASSO Cox regression analysis was performed to construct a necroptosis-related prognostic signature with four genes (BCL2, LEF1, PLK1, and BNIP3), which could serve as a prognosis biomarker in BRCA and predict the OS rate with medium to high accuracy. As far as we know, only a necroptosis-related prognostic signature has been identified for cancer (Wang and Liu, 2021). Our study was the first one to identify a necroptosis-related prognostic signature in breast cancer. Many prognostic signatures have been identified for breast cancer. Yong Shen et al. developed an immune-related lncRNA prognostic signature for breast cancer (Shen et al., 2020). Moreover, a glycolysis-related gene expression signature could be used to predict the recurrence of breast cancer (Tang et al., 2020).

Another important finding in our study was that we identified the lncRNA LINC00665/miR-181c-5p/BCL2 regulatory axis, which may play a vital role in the progression of BRCA. lncRNA LINC00665 has been proven to play a vital role in many cancers. In gastric cancer, long non-coding RNA LINC00665 was involved in tumorigenesis *via* the miR-149-3p/RNF2 axis (Qi et al., 2019). Moreover, LINC00665 could promote the progression of colorectal cancer by regulating the miR-9-5p/ATF1 axis (Zhao et al., 2020). Moreover, miR-181c-5p could mitigate carcinogenesis in cervical squamous cell carcinoma *via* GSKIP (Li et al., 2020a). Another study revealed that the inhibition of miR-181c-5p could accelerate ETM by targeting SERPINE1 in laryngeal squamous cell carcinoma (Li et al., 2020b). Our study identified the lncRNA LINC00665/miR-181c-5p/BCL2 regulatory axis in BRCA, which has never been reported before. However, further *in vivo* and *in vitro* studies should be conducted to verify this hypothesis.

## Conclusion

In conclusion, a bioinformatics method was performed to develop a prognostic necroptosis-related prognostic signature containing four genes (BCL2, LEF1, PLK1, and BNIP3) in BRCA. We also constructed a ceRNA network and identified an lncRNA LINC00665/miR-181c-5p/BCL2 regulatory axis for BRCA. Further *in vivo* and *in vitro* studies should be conducted to verify these results.

## Data Availability

The original contributions presented in the study are included in the article/Supplementary Material, further inquiries can be directed to the corresponding authors.
